# Supplementary figures and images for: Extracellular SPARC increases cardiomyocyte contraction during health and disease
Source: PLoS One. 2019 Apr 1;14(4):e0209534. doi: 10.1371/journal.pone.0209534 (PMC6443176; doi:10.1371/journal.pone.0209534)

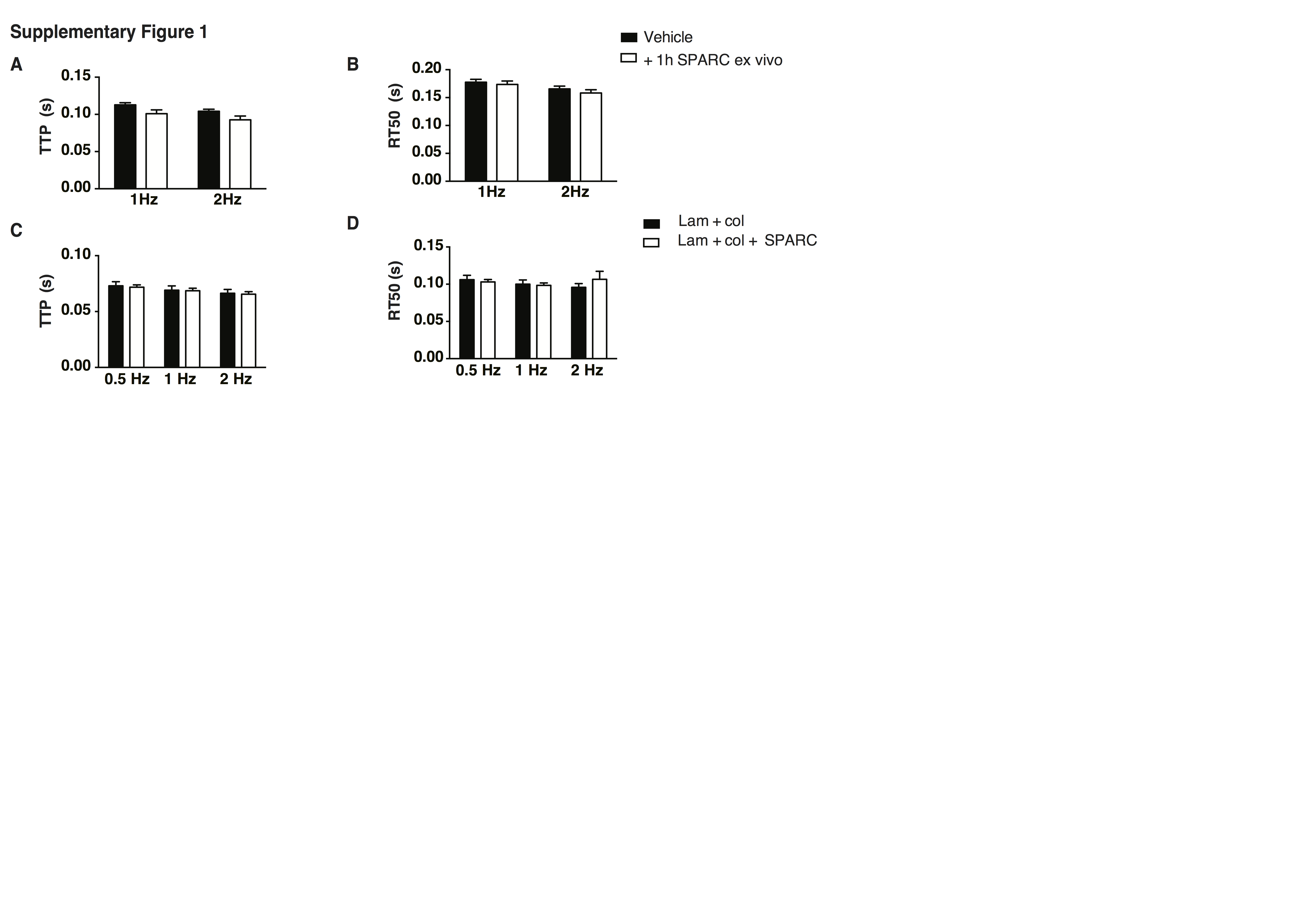

Supplement: S1 Fig — A,B Incubation of isolated adult mouse cardiomyocytes with recombinant SPARC for 1h ex vivo does not affect contraction–and relaxation times (TTP and RT50). C,D TTP and RT50 are not altered in rat cardiomyocytes grown on a matrix with SPARC. A,B N = 4 mice and >4 cells per mouse, C,D N = 3 rats and >20 cells per rat. (TIFF) [file pone.0209534.s001.tiff]

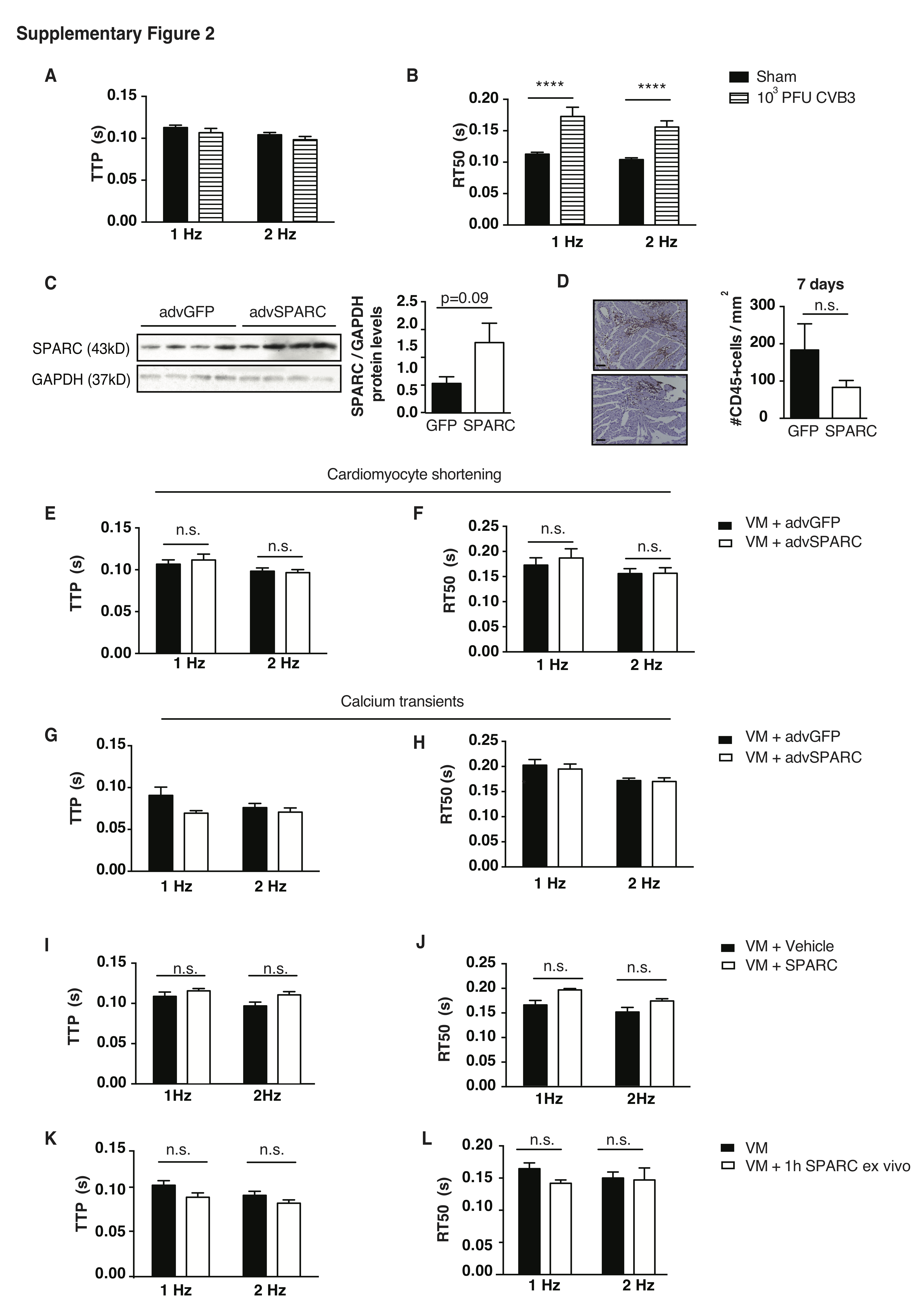

Supplement: S2 Fig — A, B Viral infection does not influence TTP but increases RT50 in isolated cardiomyocytes from virus-infected mice. C cardiac SPARC is almost significantly overexpressed in the adenoviral-SPARC injected group when compared to the control adenoviral-GFP injected mice, as shown by Western Blotting. D Slightly decreased cardiac inflammation, as measured by the amount of CD45 positive cells, was seen in the SPARC overexpressing group. E,F No effect on contraction or relaxation times was observed when SPARC was overexpressed. G,H There were no differences in the Ca2+ transient peak TTP or RT50 in cells from the SPARC overexpressing VM mice. I, J Cardiomyocytes from SPARC-treated mice demonstrated no differences in TTP or RT50. K,L When cardiomyocytes were isolated from severely sick, untreated mice, incubation of the cells with SPARC for 1h ex vivo did not influence TTP or RT50. A,B n = 11 for sham and n = 13 for VM and >3 cells per mouse, C n = 4 for both groups, D n = 5 for both groups, E-H n = 12 for advGFP group and n = 11 for advSPARC group and >3cells per mouse, I,J n = 6 for VM+vehicle and n = 7 for VM+SPARC, K,L n = 13 for both groups and >3cells per mouse. (TIFF) [file pone.0209534.s002.tiff]
