# Supplementary material for: Extracellular SPARC increases cardiomyocyte contraction during health and disease
Source: PLoS One. 2019 Apr 1;14(4):e0209534. doi: 10.1371/journal.pone.0209534 (PMC6443176; doi:10.1371/journal.pone.0209534)
Supplement: S1 Table — (DOCX) [file pone.0209534.s003.docx]

|  | **Sham**  **(n=12)** | **Mild VM**  **(n=13)** | **S** | **Severe VM**  **(n=13)** |
| --- | --- | --- | --- | --- |
| **FS (%)** | 30.59 ± 3.51 | 24.61 ± 3.50** |  | 13.41 ± 4.21**** ^####^ |
| **LVIDd (mm)** | 4.16 ± 0.20 | 4.17 ± 0.21 |  | 4.34 ± 0.22 |
| **LVIDs (mm)** | 2.89 ± 0.23 | 3.14 ± 0.23 |  | 3.76 ± 0.31****^####^ |
| **PWd (mm)** | 0.88 ± 0.15 | 0.89 ± 0.18 |  | 0.66 ± 0.15**^##^ |
| **IVSd (mm)** | 0.82 ± 0.10 | 0.78 ± 0.10 |  | 0.62 ± 0.08***^##^ |
| **HR (bpm)** | 554 ± 81 | 532 ± 50 |  | 626 ± 19**^###^ |

**p<0.01, ***p<0.001,****p<0.0001 vs. sham, ##p<0.01, ####p<0.0001 vs. mild VM

FS- Fractional Shortening, LVIDd – Left ventricular internal dimension at end -diastole, LVIDs- Left ventricular internal dimension at end systole, PWd- Posterior Wall diameter, Interventricular septum thickness at end diastole, HR-Heart Rate. Data shown ±SD
