# Supplementary material for: Extracellular SPARC increases cardiomyocyte contraction during health and disease
Source: PLoS One. 2019 Apr 1;14(4):e0209534. doi: 10.1371/journal.pone.0209534 (PMC6443176; doi:10.1371/journal.pone.0209534)
Supplement: S2 Table — (DOCX) [file pone.0209534.s004.docx]

|  | **Sham** | |  | **5 weeks VM** | |
| --- | --- | --- | --- | --- | --- |
|  | **AdvGFP**  **(n= 11)** | **AdvSPARC**  **(n=9)** |  | **AdvGFP**  **(n=17)** | **AdvSPARC**  **(n=24)** |
| **FS (%)** | 26.07 ± 3.93 | 28.65 ± 2.50 |  | 23.78 ± 5.15 | 28.58 ± 6.67 ^#^ |
| **LVIDd (mm)** | 4.00 ± 0.27 | 4.18 ± 0.17 |  | 4.02 ± 0.28 | 4.02 ± 0.20 |
| **LVIDs (mm)** | 2.94 ± 0.23 | 2.94 ± 0.20 |  | 3.03 ± 0.23 | 2.88 ± 0.29 |
| **PWd (mm)** | 0.72 ± 0.12 | 0.68 ± 0.12 |  | 0.77 ± 0.09 | 0.83 ± 0.15^#^ |
| **IVSd (mm)** | 0.74 ± 0.12 | 0.72 ± 0.10 |  | 0.73 ± 0.09 | 0.78 ± 0.10 |

#p<0.05 vs. VM + advGFP

FS- Fractional Shortening, LVIDd – Left ventricular internal dimension at end -diastole, LVIDs- Left ventricular internal dimension at end systole, PWd- Posterior Wall diameter, Interventricular septum thickness at end diastole. Data shown ±SD
