# Supplementary material for: Extracellular SPARC increases cardiomyocyte contraction during health and disease
Source: PLoS One. 2019 Apr 1;14(4):e0209534. doi: 10.1371/journal.pone.0209534 (PMC6443176; doi:10.1371/journal.pone.0209534)
Supplement: S3 Table — (DOCX) [file pone.0209534.s005.docx]

|  | **5 weeks VM**  **(prior to pump implantation)** | | |
| --- | --- | --- | --- |
|  | **V** | **VM + vehicle**  **(n=6)** | **VM + SPARC**  **(n=7)** |
| **FS (%)** |  | 14.57 ± 3.90 | 12.42 ± 4.52 |
| **LVIDd (mm)** |  | 4.49 ± 0.09 | 4.21 ± 0.21* |
| **LVIDs (mm)** |  | 3.84 ± 0.23 | 3.69 ± 0.37 |
| **PWd (mm)** |  | 0.77 ± 0.16 | 0.56 ± 0.06** |
| **IVSd (mm)** |  | 0.62 ± 0.08 | 0.61 ± 0.10 |
| **HR (bpm)** |  | 637 ± 11 | 616 ± 20 |
|  | **5 weeks VM + 72h infusion** | | |
|  |  | **VM + vehicle** | **VM + SPARC** |
| **FS (%)** |  | 12.78 ± 3.33 | 15.22 ± 4.50^#^ |
| **LVIDd (mm)** |  | 4.28 ± 0.17^#^ | 4.30 ± 0.21 |
| **LVIDs (mm)** |  | 3.73 ± 0.14 | 3.65 ± 0.37 |
| **PWd (mm)** |  | 0.71 ± 0.06 | 0.69 ± 0.11^#^ |
| **IVSd (mm)** |  | 0.68 ± 0.08 | 0.57 ± 0.08 |
| **HR (bpm)** |  | 580 ± 35^#^ | 570 ± 47 |

*p<0.05, **p<0.01 vs. vehicle sham, #p<0.05 vs. start-point

FS- Fractional Shortening, LVIDd – Left ventricular internal dimension at end -diastole, LVIDs- Left ventricular internal dimension at end systole, PWd- Posterior Wall diameter, Interventricular septum thickness at end diastole, HR-Heart Rate. Data shown ±SD
