# Supplementary material for: Extracellular SPARC increases cardiomyocyte contraction during health and disease
Source: PLoS One. 2019 Apr 1;14(4):e0209534. doi: 10.1371/journal.pone.0209534 (PMC6443176; doi:10.1371/journal.pone.0209534)
Supplement: S4 Table — (DOCX) [file pone.0209534.s006.docx]

|  | **Start (prior pump implantation)** | | |
| --- | --- | --- | --- |
|  | **Sham + vehicle**  **(n=11)** | **Sham + SPARC**  **(n=8)** |  |
| **FS (%)** | 26.14 ± 6.32 | 23.47 ± 5.12 |  |
| **LVIDd (mm)** | 3.99 ± 0.20 | 3.99 ± 0.25 |  |
| **LVIDs (mm)** | 2.95 ± 0.37 | 3.06 ± 0.32 |  |
| **PWd (mm)** | 0.64 ± 0.15 | 0.65 ± 0.13 |  |
| **IVSd (mm)** | 0.70 ± 0.15 | 0.74 ± 0.08 |  |
| **HR (bpm)** | 503 ± 19 | 519 ± 20 |  |
|  | **+ 72h infusion** | | |
|  | **Sham + vehicle**  **(n=11)** | **Sham + SPARC**  **(n=8)** |  |
| **FS (%)** | 26.80 ± 4.92 | 34.73 ± 7.57*^#^ |  |
| **LVIDd (mm)** | 4.05 ± 0.16 | 4.13 ± 0.17 |  |
| **LVIDs (mm)** | 2.97 ± 0.28 | 2.67 ± 0.33*^#^ |  |
| **PWd (mm)** | 0.74 ± 0.09^#^ | 0.84 ± 0.12^#^ |  |
| **IVSd (mm)** | 0.74 ± 0.09 | 0.83 ± 0.12 |  |
| HR (bpm) | 524 ± 54 | 556 ± 36 |  |

*p<0.05 vs. vehicle sham, #p<0.05, ##p<0.01 vs. start-point

FS- Fractional Shortening, LVIDd – Left ventricular internal dimension at end -diastole, LVIDs- Left ventricular internal dimension at end systole, PWd- Posterior Wall diameter, Interventricular septum thickness at end diastole, HR-Heart Rate. Data shown ±SD
